# Structural and Dynamical Properties of Nucleic Acid Hairpins Implicated in Trinucleotide Repeat Expansion Diseases

**DOI:** 10.3390/biom14101278

**Published:** 2024-10-10

**Authors:** Feng Pan, Pengning Xu, Christopher Roland, Celeste Sagui, Keith Weninger

**Affiliations:** 1 Department of Physics, North Carolina State University, Raleigh, NC 27695, USA; fpan3.ncsu@gmail.com (F.P.); cmroland@ncsu.edu (C.R.);; 2 Department of Statistics, Florida State University, Tallahassee, FL 32306, USA; 3 Department of Pharmacology, University of North Carolina at Chapel Hill, Chapel Hill, NC 27599, USA

**Keywords:** trinucleotide repeats, expansion diseases, hairpin structure, single-molecule FRET, smFRET, molecular dynamics simulations, CAG and GAC, CTG and GTC

## Abstract

Dynamic mutations in some human genes containing trinucleotide repeats are associated with severe neurodegenerative and neuromuscular disorders—known as Trinucleotide (or Triplet) Repeat Expansion Diseases (TREDs)—which arise when the repeat number of triplets expands beyond a critical threshold. While the mechanisms causing the DNA triplet expansion are complex and remain largely unknown, it is now recognized that the expandable repeats lead to the formation of nucleotide configurations with atypical structural characteristics that play a crucial role in TREDs. These nonstandard nucleic acid forms include single-stranded hairpins, Z-DNA, triplex structures, G-quartets and slipped-stranded duplexes. Of these, hairpin structures are the most prolific and are associated with the largest number of TREDs and have therefore been the focus of recent single-molecule FRET experiments and molecular dynamics investigations. Here, we review the structural and dynamical properties of nucleic acid hairpins that have emerged from these studies and the implications for repeat expansion mechanisms. The focus will be on CAG, GAC, CTG and GTC hairpins and their stems, their atomistic structures, their stability, and the important role played by structural interrupts.

## 1. Introduction

The stability of the genome is important to all organisms in order to maintain the complex network of biochemical interactions that underlie life. The survival of any organism is challenged by exposure to agents that damage the genome, be they external agents (such as UV light, ionizing radiation, and toxic chemicals) or harmful products of endogenous metabolic processes (such as reactive oxygen). Such genome-modifying agents can benefit humans, for example when used as anti-microbials (e.g., industrial sterilization of materials by gamma-ray exposure), but they can also result in human diseases with cancer being most prominent.

Beyond these sorts of external stressors, it has been recognized that another mutagenic agent is encoded in the DNA sequence itself: nonstandard or atypical nucleic acid structures that can result from certain DNA sequences [1]. One of the most significant classes of sequence motifs associated with genomic instability is simple sequence repeats (SSRs) consisting of one to six (and even 12) nucleotides that are repeated up to 30 times (and more for pathological cases) [2]. In many cases, these repeated sequences in the genome are surprisingly susceptible to changes in their length.

Some instability of SSRs can be part of the natural processes in cells. It has been estimated that the rate of length mutations in some SSRs is about 10^5^ times higher than that of a point mutation [3]. This can lead to frequent polymorphism in the coding regions of the genes with a correspondingly rapid expansion of the amino acid repeats. This variability aids natural selection by rapidly generating new alleles and facilitating evolution.

In addition to these productive mutations, SSRs may also exhibit “dynamic mutations” that do not follow Mendelian inheritance. About a century ago, in humans, it was observed that the neurological myotonic dystrophy disorder [4,5] was an inherited disease whose age of onset decreased and whose severity increased with successive generations. Many other similar human disorders joined the family of so-called “genetic anticipation” diseases, but it took scientists until the 1990s to discover that these diseases were caused by the intergenerational expansion of SSRs [6,7,8]. To date, approximately 50 DNA expandable SSR diseases have been identified, and the list is expected to grow [9,10]; see Table 1. Despite being involved in dozens of diseases, the mechanisms underlying the instability of SSRs are generally unconfirmed, and the connections between the change in the repeat sequence and the emergence of disease phenomena are similarly opaque. In this review, we address recent progress toward uncovering mechanisms for a subset of human diseases known as trinucleotide repeat expansion diseases.

### 1.1. Trinucleotide Repeat Sequences and Associated Human Diseases

Trinucleotide repeats (TRs) constitute the most common type of SSRs in the exome of all known eukaryotic genomes [12,13,14,15]. RNA transcripts containing TRs may greatly outnumber their genomic templates [16]. TRs in DNA may be neutral sequences or have several regulatory roles in gene expression. In RNA, they are believed to play a regulatory role mediated by interactions with TR binding proteins [17,18,19], regulating splicing, maturation and transport [20,21,22,23,24,25,26,27], while in the cytoplasm, they may regulate mRNA stability and translation [28] or promote repeat-associated non-AUG (RAN) translation [29,30]. The incidence of specific sorts of TRs and their position in genes varies greatly between different genomes, underscoring the vital role of TRs in genome evolution [12,31]. The dynamic changes in the length of TR regions in human genes cause disorders known as Trinucleotide (or Triplet) Repeat Expansion Diseases (TREDs) [32,33,34]. Triplet expansion diseases are a group of genetic disorders that include diseases such as Huntington’s disease, Myotonic dystrophy, spinocerebellar ataxia, and fragile X syndrome that affect the function of nerve cells, leading to severe neurological problems [8,35].

The genomic instability underlying Huntington’s disease provides an example of trinucleotide repeat expansion phenomena. Huntington’s disease pathology emerges when the CAG repeat sequence in the huntingtin protein gene expands from the typical 17–23 repeat range across a threshold of 36 repeats [36,37,38,39]. The expanded CAG region will result in mRNA containing the expanded region as well as a longer polyglutamine insert within the translated huntingtin protein. Although these expanded polyglutamine regions make the protein more susceptible to aggregation and induce plaques, exactly what processes are toxic and lead to cell death remains under investigation [40,41,42,43,44].

**Figure 1:**
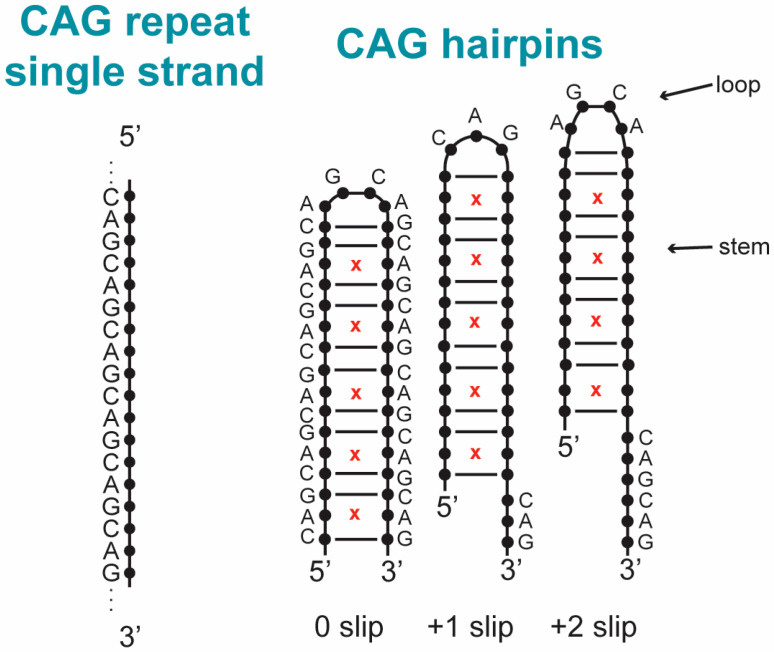
Examples of hairpins formed from CAG-repeat containing DNA. A single strand of CAG repeated DNA (**left**) can fold on itself to form hairpins (**right**). The stem is a duplex where a third of the base pairs are A–A mismatches (marked by red x) and the loop at the end includes unpaired bases. Three different states of the hairpin are displayed that are slipped in steps of a CAG unit indicated below the hairpin (leaving a short single strand overhang in this example). Some base labels are omitted for clarity. The stem base pairing pattern is maintained with slipped states. The loops illustrate that even and odd numbers of repeats in the stem result in tetraloops or triloops with 4 or 3 unpaired bases respectively.

More fundamentally, the processes causing the CAG expansion in the genome are unknown. The expansion is believed to be primarily caused by some sort of slippage during DNA replication, repair, recombination or transcription [1,8,9,10,32,33,34,45,46,47,48,49,50,51,52,53], which involves the transient separation of complementary DNA strands or exposure of a single DNA strand. One important breakthrough has been the recognition that all expandable repeats have atypical structural characteristics [8,10,47,53,54], including single-stranded hairpins, Z-DNA, triplex, G-quartets and slipped-stranded duplexes. In the example of Huntington’s disease, models of mechanisms of expansion are focused on hairpins that can result when the CAG repeat-containing single-strand DNA folds on itself (Figure 1) [50,54,55,56,57]. Such DNA hairpin structures formed from a trinucleotide repeat have a duplex-type stem with 2/3 correct base pairing and 1/3 mismatched bases and a loop of three or four bases at the turn (Figure 1). These structures are likely recognized by mismatch repair proteins with their subsequent enzymatic processes starting a chain of events that result in the expansion. Cell toxicity and death arise in cells that contain expansions by pathways associated with toxic gain-of-function or loss-of-function for RNA transcripts and translated proteins [9,19,58,59,60,61,62,63,64,65,66,67]. The precise connection between TR expansion and pathological processes in cells remains a point of debate that we do not address in this review [41,42]. Rather, our focus here is on processes that relate to the expansion of the TR in the genome.

### 1.2. DNA Mismatch Repair Proteins Are Causative for Disease-Related Expansion of Trinucletoide Repeat Sequences

How exactly do atypical structures manage to “gum up the works” and lead to proliferated expansion? After all, do cells not have the capability to repair these kinds of errors? This is a largely unknown, open-ended question very much at the forefront of current research. The DNA mismatch repair (MMR) system acts after DNA replication and recombination to maintain genomic stability by recognizing and repairing chemically modified bases, mismatched base pairs, or insertion/deletion loops [68,69,70,71,72]. Perplexingly, the action of MMR has been associated with TR expansion [36,52,73,74,75,76,77,78]. In eukaryotes, MMR is initiated by either one of two heterodimeric MutS homolog complexes, MSH2-MSH6 (MutSα) and MSH2-MSH3 (MutSβ), which combine lesion binding with ATP hydrolysis with distinct but sometimes overlying specificities [79,80]. MutSα repairs single-base mismatches and small, one or two-nucleotide loops. MutSβ has strong specificity for two- to fifteen-nucleotide loops but can also signal the repair of some single-base mismatches. Mutations in the genes encoding the proteins of the MMR system can lead to further mutations and microsatellite instability throughout the genome [68,69,70,72], as is the case of hereditary non-polyposis colorectal cancer [74,81,82,83,84,85,86,87,88,89]. In contrast, MMR machinery that is not defective itself can facilitate different outcomes when encountering some types of DNA damage, where the process of lesion binding can trigger processes leading to cell-cycle checkpoint or apoptosis activation [72,90,91,92,93,94]. In the case of encountering TR duplexes, the MMR system most alarmingly becomes one of the causative factors leading to TR expansion [52,53,73,78]. These instabilities are localized only in the TRED allele, and do not spread throughout the genome as occurs with mutated MMR proteins. Genome-wide association studies (GWAS) correlated variants in MMR proteins to changes in CAG expansion disease severity in Huntington’s disease patients [76,95,96,97], and patient-derived stem cell genetic manipulations further extended these conclusions [98]. In particular, the crucial role of MMR proteins in TR expansion has been shown in transgenic mouse models of Huntington’s disease [99,100,101] and other expansion diseases [102,103,104]. In crosses of Huntington’s disease (HD) transgenic mice with mice lacking MSH2, the CAG expansion was attenuated in both the somatic tissue of their descendants and in their germ cell [105,106]. In transgenic mice, the absence of MSH3 suppresses the expansion of the CTG repeat in the 3′ non-coding region of the DM1 transgene as well as the expansion of the CAG repeat in the coding sequence of the HD transgene [52,107]. Instead, the loss of MSH6 has increased somatic repeat instability [108], which suggests that MutSα may try to prevent TR expansion. Findings of high levels of TRs and MSH3 in post-mitotic neurons in mice and humans also supported the notion that high levels of MutSβ can cause TR expansion in nondividing cells [109].

While the workings of the MMR system are quite complex and the mechanisms leading to TREDs are still fairly unknown, there are associated features that are robust. In most TREDs, there is a correlation between the number of repeated nucleotides beyond a critical threshold and their increased further expansion and subsequent increased pathology. Thus, the age of disease onset decreases while the disease severity increases with each successive generation as the number of nucleotide repeats continues to increase. Another important breakthrough in the understanding of TREDs has been the recognition that the critical step in all models of repeat instability is the stable or transient formation of atypical non-B DNA stable secondary structures in the expandable repeats [32,46,55,78,109,110,111,112] and that these non-B DNA conformations “*not the sequences per se, promote mutagenesis in flanking regions*” [112]. While there are a number of pathogenic structures known to be associated with TREDs, i.e., hairpins, triple helices, Z-DNA, G-quadruplexes and others, it is the hairpin structures based on single-stranded DNA (ssDNA) that are by far the most prolific and commonly associated with TRs. Thus, an important first step in understanding the complex cascade of molecular changes undergone by the MMR system is to probe the structural and dynamical characteristics of nucleic acid hairpins.

In this brief review, we summarize the results of recent experimental and theoretical studies of SSR-based secondary structures implicated in TREDs; we will feature our own work based on large-scale molecular dynamics (MD) simulations [113,114,115,116,117,118,119,120,121,122] and single-molecule Fluorescence Resonance Energy Transfer (smFRET) [11,123,124] (for smFRET reviews, see Refs. [125,126]). Primarily, we shall discuss the structure and dynamics of DNA/RNA hairpins of the most common TRs; these include DNA/RNA CAG, GAC, CTG (CUG for RNA), GTC (GUC for RNA), CCG, and GGC.

## 2. Results

For TREDs, the most common pathogenic structures are hairpin loops. Until recently, relatively little was known about the structure and dynamics of these loops at the molecular level. However, recent smFRET and nuclear magnetic resonance (NMR) experiments complemented by classical MD simulations are starting to reveal their atomistics, which we review here along with implications for SSR expansion mechanisms. For presentation purposes, it is convenient to discuss the results in terms of the homoduplexes representing the hairpin stem and the actual loop parts separately.

**Figure 2:**
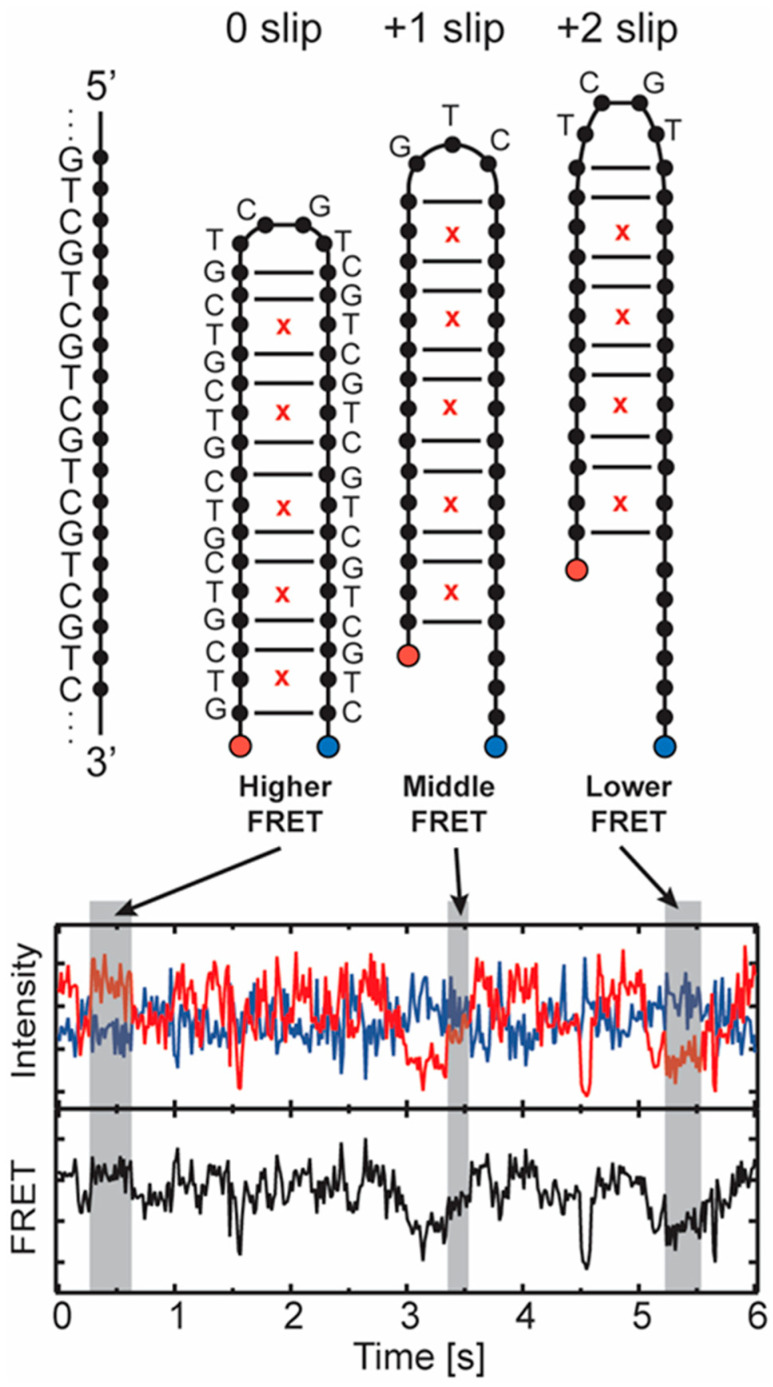
Example of smFRET assay for detecting slipped states of trinucleotide repeat DNA hairpins. On the left, a single-strand GTC repeat DNA is illustrated. To the right, 3 slipped states of the GTC folded into a hairpin are displayed. The acceptor on the 5′ end is shown in red, and the donor is shown in blue. T–T mismatches are shown as red x. The sequence in the loop is shown to illustrate tetraloop and triloop states that associate with the parity of the slipped state, but the stem sequence is not shown for the +1 and +2 slipped states for clarity. The different slipped states result in different donor–acceptor separations and different FRET values as indicated. The example measured data trace (**lower**) shows donor intensity (blue), acceptor intensity (red), and FRET efficiency ratio (black) vs. time. See Refs. [123,124] for more information.

The experimental results discussed below were primarily generated using smFRET measurements. For these studies, DNA containing different numbers of triplet repeat units for several distinct sequences was tethered to a passivated surface by a biotin–streptavidin interaction. The triplet repeat-containing DNA folds on itself into a hairpin configuration. The hairpin includes an acceptor fluorophore on its free end, and a donor is attached at a fixed position on the surface anchoring strand (Figure 2, blue and red dots). The distance between the donor and acceptor changes when the hairpin slips along its axis, allowing the FRET coupling to report the configuration of the hairpin. A prism-type total internal reflection single molecule fluorescence microscope [126] records the signals [123]. An example of data from a GTC hairpin is shown in Figure 2, where anticorrelated intensities from the donor and acceptor result from the spontaneous slipping of the hairpin. Studies of this sort, using different triplet repeat sequences, characterized the sliding dynamics of hairpins in repeat units, and quantitative analysis of the kinetics [127] can reveal the energetics of the process.

The computational results are based on atomistic Molecular Dynamics (MD) and on our Adaptively Biased Molecular Dynamics (ABMD) method [128,129] (an umbrella sampling approach with a time-dependent potential), which has been combined with generalized Replica Exchange Molecular Dynamics [130] (REMD) along with a Steered Molecular Dynamics [131] procedure (SMD). The resulting codes allow for the efficient evaluation of biomolecular free energies and sampling of conformational space. These procedures ultimately can yield highly accurate free energy curves and equilibrium properties for trinucleotide repeat hairpins. The MD simulations can reveal details of the atomic configurations of the loops of the hairpins and elucidate how these configurations relate to the stability of the structures. The combination of smFRET and molecule dynamics studies help us understand how these hairpin structures contribute to populating states associated with trinucleotide expansions and ultimately connect to the pathogenesis of the related diseases. We explore details of the combination of these investigations below.

### 2.1. Hairpins Formed from CAG and GAC TRs

CAG TRs give rise to the largest group of neurodegenerative diseases, which may be a reflection of the fact that CAG tracts are overrepresented in the human genome. Thus, studies show [132] that CAG tracts of six or more repeats occur 1055 times in the human genome with 300 such tracts in the exons. CAG tracts of ten or more occur 136 times with 33 tracts in the protein-coding region. These numbers are to be contrasted with those associated with GAC tracts: these occur only sixteen times in the human genome with three in the exon region. The specific TRED expressed depends very much on the location of the CAG tract within a given gene. Thus, CAG repeats in the 5′-UTR region of the PPP2R2V gene give rise to spinocerebellar ataxia type 12, while CAG tracts in the exon are responsible for nine neurodegenerative disorders such as Huntington’s disease (HD), bulbar and spinal atrophy (SBMA), dentatorubralpallidolysian atrophy (DRPLA) and several different spinocerebellar ataxias (SCAs). Generically, CAG repeats give rise to what is known as polyglutamine (polyQ) diseases [133], as these tracts all give rise to polyQ expansions. These diseases are associated with a critical TR number for the pathological expansions. For example, in HD, a repeat number less than about 34 is considered normal, while repeats in the 36 to 250 range are pathological, leading to disease expression. Although the pathologies of the CAG-associated TREDs are different, they all give rise to polyQ aggregates with cross-beta conformations ultimately associating with neuronal death [134,135].

It is interesting that GAC TR repeats are also associated with a specific set of diseases that are different from the CAG-associated TREDs. Unlike CAG expansion diseases that often involve the growth of CAG repeat tracks by tens to hundreds of CAG units, GAC diseases are associated with only small changes in the TR repeat number. Multiple epiphyseal dysplasia is caused by a one or two-unit increase in the (GAC)_5_ tract within the gene for the human cartilage oligomeric matrix protein to a (GAC)_6_ or (GAC)_7_ tract [136]. In contrast, within the same gene, contraction of the (GAC)_5_ tract by one GAC unit to (GAC)_4_ causes pseudoachondroplasia [137,138]. It turns out that the specific structure of the GAC duplexes is dependent on the pH and the ionic strength [139]. As will be discussed later, experimental studies show that the loop dynamics associated with CAG and GAC are very different, which gives rise to their contrasting behavior and may relate to differences in disease phenomena.

#### 2.1.1. CAG and GAC Homoduplexes

**Figure 3:**
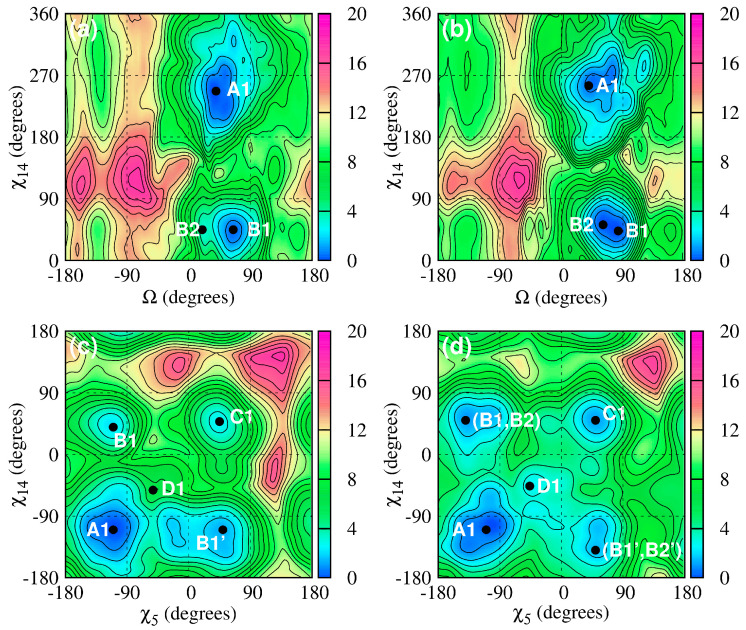
Shown here are sample free energy landscapes for a single A–A mismatch in a DNA-CAG and DNA-GAC. Here, (**a**) is (χ,Ω) for DNA-CAG; (**b**) (χ,Ω) for DNA-GAC; (**c**) (χ,χ) for DNA-CAG and (**d**) (χ,χ) for DNA-GAC. The letters mark the most important local minima with associated structures shown in Figure 4. The primed letters represent minima that are approximate mirror images of unprimed minima. Here, collective variable χ represents a dihedral angle of a given nucleotide and Ω an angle probing the motion of the nucleotide outside of its helical core. See Ref. [117] for more details.

**Figure 4:**
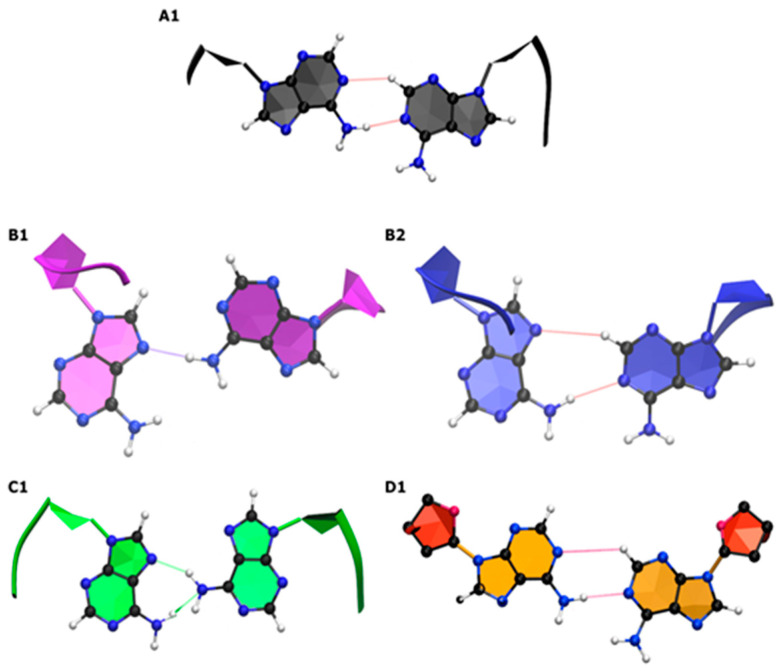
Shown here are sample A–A mismatch conformations associated with the primary minima on the free energy landscapes of Figure 3 with letters denoting different conformations (indicated on Figure 3). Here, (**A1**) is associated with anti–anti; (**B1**,**B2**) with syn–anti; (**C1**) with syn–syn; and (**D1**) is a special case in which the *χ*-angle is syn-syn, but the base conformation appears anti-anti given the sugar ring twisting to be parallel to the bases. Hydrogen bonds associated with the configurations are marked. For more details, please see Ref. [117].

The behavior of the A–A mismatches in CAG and GAC homoduplexes determines the homoduplex structures. In CAG TRs, the Watson–Crick base pairs between the mismatches exhibit GpC steps, while in GAC TRs, the corresponding steps are CpG. MD simulations based on the AMBER simulation package [140] and augmented with special free energy methods (ABMD) [129] with carefully chosen collective variables show distinct characteristics of the homoduplexes [117,141]. Figure 3 and Figure 4 display CAG and GAC-based free energy landscapes and corresponding configurations of nucleotides associated with the main minima. The free energy calculations show that the preferred duplex structure is characterized by A–A mismatches stacked inside the helical core with the nucleotide torsion angles in an anti–anti conformation for both RNA and DNA. This corresponds to torsion angles of about 180–200° degrees for RNA and high-anti—about 230–260° degrees—for DNA. Beyond this, the next most favorable conformation corresponds to nucleotides in an anti–syn configuration, which is followed by nucleotides in their syn–syn conformation. Sample free energy landscapes based on the torsion angle (χ) and a collective variable (Ω) probing for the swinging out of nucleotides from their helical core are shown in Figure 3, while Figure 4 shows corresponding sample conformations. It should be noted that for nucleotides in a given torsion angle conformation, several different hydrogen bond structures are possible and have been characterized [117]. These results are consistent with experimental studies on CAG-RNA [141]. We note that the differences between the DNA and RNA anti-conformations are due to the presence of an additional hydroxyl group associated with the RNA sugars. This hydroxyl group interacts with the RNA backbone in such a way as to reduce the range of torsion angles explored by the RNA homoduplex.

The dynamics of CAG and GAC homoduplexes was characterized via the Principal Component Analysis (PCA) [142]. Large fluctuations have been observed for DNA in its global minimum structure, corresponding primarily to a coupling between the bending and unwinding modes of the helix. Similar fluctuations were found for the A-RNA, but to a lesser degree due to the already noted coupling between the sugar hydroxyl and backbone. Transitions between different nucleotide conformations, e.g., anti–syn to anti–anti, have also been investigated. These kinds of transitions involve local distortions around the A–A mismatches and involve combinations of base flipping, base stacking and base rotation in either the major or minor grooves [117]. Both CAG and GAC DNA homoduplexes experience some degree of unwinding with CAG unwinding occurring at the mismatch and GAC unwinding taking place at the CpG steps. There is, however, no evidence for the formation of transient left-handed helical structures such as Z-DNA.

#### 2.1.2. Structure and Dynamics of CAG and GAC Loops

There is a delicate interplay between the free energies of the loop and stem parts of a TR hairpin which determines its behavior. Recently, it has become possible to probe this behavior by means of combined smFRET experiments and MD simulations [11,123,124]. smFRET was used to probe the parity-dependent slipping behavior in CAG and GAC hairpins, which has important ramifications for the loop structure. In the experiments, a two-stranded system was used involving an anchor and a hairpin strand with acceptor and donor fluorophores placed in suitable, consistent positions such that when the donor and acceptor come near each other, FRET signals of differing intensities are measured (Figure 2 for examples). This in turn signals the opening, closing, and slipping of the hairpin, and its kinetics may be inferred [123,124].

To probe CAG hairpin slippage, two hairpin structures (CAG)_14_ and (CAG)_15_ with an even and odd parity were constructed [123,124], and time traces of their FRET signals were analyzed [127]. See Figure 1 and Figure 2 for the schematics associated with the general idea of this parity behavior and sample FRET data. For (CAG)_14_, three FRET states of varying efficiencies—0.01, 0.31, and 0.65—were observed. The 0.01 state is rarely visited. It could arise from opening of the hairpin or blinking of the acceptor fluorophore. The populations for the 0.31 and 0.65 states are about 20% and 80%, respectively. Turning to (CAG)_15_, four different states with FRET efficiencies of 0.01, 0.25, 0.46 and 0.73 were observed. Again, populations for the open state (0.01) were negligible, while for the other states, they were estimated to be 5% for 0.25, 60% for 0.46 and 30% for 0.73, respectively. Subsequent analysis showed that the 0.73 state for (CAG)_15_ is similar to the 0.65 state for (CAG)_14_ and corresponds to the donor and acceptor fluorophores moving closer together by a single CAG unit, or a −1 slip. Likewise, the 0.46 state is associated with the hairpin slipping by one CAG unit away from the donor, corresponding to a +1 slip. So clearly, there is back and forth slippage for both strands by a single CAG TR unit. Structurally, for (CAG)_15_, this allows for the formation of a 5′-AGCA-3′ tetraloop with CAG/GAC aligned pairing for the duplex stem. Contrast this with a hairpin with the ends CAG/GAC aligned, i.e., 0 slip. This, in turn, leads to a triloop 5′-CAG-3′ structure consisting of a single CAG unit. The smFRET and simulation results indicate that these kinds of loops are less stable than the tetraloops. Analyzing the remaining data in a similar fashion indicates that the 0.31 state of (CAG)_14_ may be associated with slippage via +2 CAG units, and the 0.25 state of (CAG)_15_ may be associated with a +2 unit shift [123,124].

This behavior turns out to be robust and forces an even/odd repeat parity onto hairpin slippage. Hairpins with an even number of repeats form a 5′-AGCA-3′ tetraloop with a stem fully paired or slipped by two CAG units. By contrast, a hairpin with an odd number of repeats and a fully paired stem is associated with a 5′-CAG-3′ triloop. Such conformations, however, are less stable, and the hairpin slips back and forth to form a 5′-AGCA-3′ tetraloop with a hanging CAG TR unit in the stem. This differing slippage behavior between hairpins with even and odd repeat numbers illustrates nicely the interplay between loop and stem structure and agrees with the MD simulation results [123,124].

**Figure 5:**
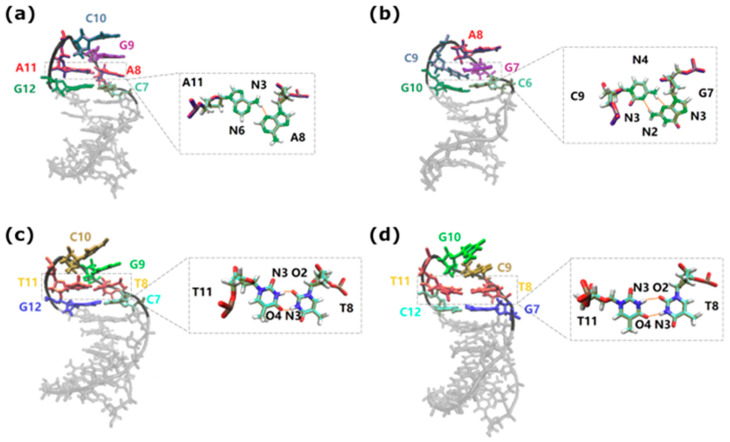
(**a**) A CAG tetraloop with the hairpin part in A(anti)–G(anti)–C(anti)–A(syn) form, with inset showing the hydrogen bond A8:N3-A11:N6 in the closing A–A mismatch. (**b**) GAC triloop with the hairpin part of G(anti)–A(anti)–C(syn) form, with inset showing the hydrogen bond G7:N2-C9:N3 and G7:N3-C9:N4 in the closing CG pair. (**c**) CTG tetraloop with the hairpin part in T(anti)–G(anti)–C(anti)–T(anti) with inset showing the hydrogen bond T8:N3-T11:N4 and T8:O2-T11:N3 in the closing T–T mismatch; (**d**) a GTC tetraloop with the hairpin part in T(anti)–G(anti)–C(syn)–T(anti) with inset showing the hydrogen bond T8:N3-T11:N4 and T8:O2-T11:N3 in the closing T–T mismatch. For more information, please see Refs. [123,124].

MD simulations have allowed for the direct study of the loop atomistics, which in turn explains their differing stabilities. Figure 5 shows sample loop configurations. The CAG triloop has nucleotides in their anti-conformation with the C-base being flipped out forming a sheared C–G pair characterized by a single hydrogen bond. The loop is then ‘held together’ by a weak AG/CA step with mismatched A’s. The 5′-AGCA-3′ tetraloop is more stable as it is held together by favorable stacking energy within the loop. There is also reduced bending and deformation of the backbone with the base held together by a locking CG/GC step. There is also more flexibility in terms of the base conformations of the loop; the AGCA nucleotides may be in either an anti–anti–anti–anti or an anti–anti–anti–syn conformation. In turn, these conformations display populations with a differing number of single and double base stacks within the loop [123]. How does the transition from tri- to tetra-loop take place? The simulations suggest that such transitions are triggered through the disruption of the A–A mismatch closest to the loop. The A base on the 3′ strand turns toward the minor groove, forming a transient GACG tetraloop, which presumably is the first step toward the formation of a more stable tetraloop. Importantly, CAG hairpins within a configuration of a three-way junction where the hairpin occupies one arm and the other two arms are CAG-CTG DNA duplexes that permit short migrations of the CAG hairpin [143,144,145] display similar trends as the simple hairpins described above. The significance of the loop region on the stability of CAG hairpins is further confirmed by smFRET studies of CAG hairpins where the loop region is replaced by poly-A or poly-T linkers. These new sequences did not display the characteristic slipping behavior but rather transitioned between simple fully open and fully closed hairpin conformations [146].

Having understood the basics of CAG slippage, it is interesting to probe how interrupts or mutations change both loop structure and dynamics. This is not just an academic question, as mutations and interrupts strongly influence disease expression. For instance, the point mutation G → A which changes CAG to CAA (both coding for the same amino acid glutamine) is known to have a stabilizing effect on HD [147,148], SCA17 [149], and SCA2 [150,151,152]. Mechanisms of how trinucleotide interrupts lessen TRED phenomena have been suggested to relate to stabilizing the dynamics of hairpins [153]. To explore the effects of CAA interrupts on CAG hairpin dynamics, smFRET experiments and MD simulations have been used to study (CAG)_6_(CAA)(CAG)_8_ and (CAG)_7_(CAA)(CAG)_8_ hairpins [11,123] in which the CAA interrupt is placed at or near the loop position. The experiments show that in the case of the former, the +1 slip is dramatically preferred for the former and the −1 slip is preferred for the latter. Both of these results are consistent with a tetraloop sequence of AACA closed by two G–C Watson–Crick bonds. Overall, the CAA interrupts in CAG TRs dramatically reduce strand slippage in the hairpins. MD simulations results are completely consistent with the experimental results. Essentially, for the G → A mutation, which changes the 5′-AGCA-3′ tetraloop to 5′-AACA-3′, the base stacking is preserved, and the stability considerations are not dramatically altered. However, placing the mutation elsewhere in or around the loop either adds mismatches or destabilizes the stacking, which ultimately gives a less stable hairpin. Similar stabilization has been observed with smFRET for CAA interrupts in CAG hairpins in the context of three-way junctions [143].

Other mutations have also been explored, and the results are briefly summarized here [123,124]. Experimentally, changing the middle CAG → AAA in (CAG)_15_ ultimately gives rise to a dominant AAA triloop. MD simulations agree with these results, which may be understood in terms of stronger purine–purine (A/A) stacking over the pyrimidine–purine (C/A) stacking of CAG-based triloops. Other explored variants consider mutations of CAG → CGG, CAG → CAC, and CAG → CTG. It is interesting to note that the former two mutations are in favor of positioning the mutated nucleotide around the loop, whereas the CAG → CTG mutation favors the configuration that avoids the hairpin loop and puts the thymine closer to the hairpin stem. In terms of CAG → CGG, smFRET experiments on (CAG)_6_(CGG)(CAG)_8_ are characterized by +1 or −1 slips, which is indicative of GGCA or AGCA tetraloops, respectively. The GGCA is favored over the AGCA loop in the experiment, although comparison is difficult because the alignments of bases in the stem are different, too. MD simulations support the stability of the GGCA tetraloop. Results based on (CAG)_6_(CAC)(CAG)_8_ hairpins show that an ACCA tetraloop is favored with this mutant. However, shifting the mutation around can lead to more complicated behavior with the possible formation of larger loops [123,124].

smFRET has also been used to study GAC hairpins [124], and its behavior is considerably different from that of the reverse sequence CAG hairpins. Both (GAC)_14_ and (GAC)_15_ hairpins were investigated, and the results show that GAC hairpins are most commonly found in a triloop configuration with slips of +1 or −1 being the most common. MD simulations show that the 5′-CAG-3′ triloop prefers anti–anti–syn conformations for its hairpin. The hairpin is stabilized by the G/A stacking of the loop bases and a sheared G_anti_–C_syn_ base pair with two hydrogen bonds. By contrast, the 5′-ACGA-3′ tetraloop is characterized by fluctuations between a number of closely related states. The MD simulations indicate a preference for the anti–anti–anti–syn conformation with one persistent hydrogen bond in the A_anti_–A_syn_ pair. The state has two possible conformations with either no stacking in the loop or one with a two-stack A/C and G/A state. The propensity of GAC to form triloops has also been observed in previous experimental studies based on polymerase extension [154]. The contrasting CAG and GAC hairpin structures likely influence the different expansion propensities of their repeated tracts seen in distinct diseases [41,42,136], but the mechanisms connecting these expansions to disease states remain opaque.

### 2.2. Hairpins Formed from CTG (CUG for RNA) and GTC (GUG for RNA) Repeats

Turning to the structure and dynamics of CTG expansions (CUG in RNA), we note that these expansions give rise to a number of TREDs, while GTC (GUC in RNA) do not exhibit such pathological expansions. In particular, myotonic dystrophy begins in adulthood [155,156] and is caused by CTG TRs (myotonic dystrophy type 1) or CCTG (myotonic dystrophy type 2) expansions. Thus, CTG expansions in the 3–38 range are considered normal, 39–50 repeats premutation are considered abnormal, and 50+ repeats lead to disease expression. For CTG, the TRs are located in the 3′-UTR of the dystrophia myotonic protein kinase gene; CCTG repeats are associated with the zinc finger (ZNF9) gene [157]. There is considerable evidence that d(CTG) and r(CUG) form hairpin structures with the latter being associated with a toxic mRNA gain-of-function [23]. Experimentally, RNA CUG TRs are known to form homoduplexes in an A-RNA form dominated by their U–U mismatches. These are quite dynamic associated with relatively large fluctuations and are therefore known as “wobble U–U” mismatches [158,159,160,161].

#### 2.2.1. CTG (CUG for RNA) and GTC (GUC for RNA) Homoduplexes

The structure and dynamics of d(CTG)/r(CUG) and d(GTC)/r(GUC) homoduplexes have been investigated both experimentally [158,159,160,161] and theoretically with MD simulations [119,162]. For d(CTG)/r(CUG), the mismatches are separated with a Watson–Crick base pair GpC step, while CpG steps separate mismatches in d(GTC)/r(GUC). The studies show that the global free energy minima associated with the T–T and U–U mismatches correspond to anti–anti conformations inside the helical core. While both mismatches are very dynamic, the fluctuations associated with the U–U mismatch are larger than the T–T fluctuations. The mismatches themselves are characterized by structures with the hydrogen bond number varying between zero and two. Beyond the global minimum, the next minima correspond to anti–syn or syn–anti conformations followed by syn–syn. As with CAG/GAC TR structures, the anti–anti U–U conformations in r(CUG)/r(GUC) duplexes are within their regular range, while those corresponding to d(CTG)/d(GTC) are in the “high-anti” range [119].

Dynamical simulations of the homoduplexes show that the T–T and U–U mismatches can flex between a large number of conformations without significantly altering the global helical structure. PCA analysis of the fluctuations of the first eigenvector around the backbone displays a coupling between bending and unwinding modes, which is stronger in the RNA helices. Dominating the mismatched base-pair dynamics are opening, shear and stretching modes. For RNA, there is also a widening of the major groove with under twisting. There is also a decrease in the inclination angle with respect to the standard A-RNA and B-DNA structures [119].

#### 2.2.2. Structure and Dynamics of CTG and GTC Hairpins

CTG and GTC hairpins have been studied with smFRET in a manner similar to the CAG and GAC hairpins as discussed above [124,163]. Additionally, CTG has also been investigated with Nuclear Magnetic Resonance (NMR) [164], and a unified picture of the hairpin structure has emerged. Perhaps not surprising, the structure and behavior of CTG loops resemble those of CAG loops, while GTC loops are more like those with GAC. There are, however, some differences.

A pattern emerges in which CAG, CTG and GTC prefer tetraloop configurations with GTC being more tolerant of triloops; GAC, as already mentioned, prefers a triloop configuration. Both CTG and GTC display parity-dependent behavior based on strand length. Here, we simply give some of the results [124]: (CTG)_14_ hairpins spent 85% of the time in a 0 slip configuration indicative of a tetraloop, 2% of time in a −2 slip also indicative of a tetraloop and only 4% in a +1 slip consistent with a triloop. Similarly, for (CTG)_15_, it was observed that hairpins were characterized by slips of +1 (33%) and −1 (57%) consistent with tetraloop structures and only 7% in a triloop configuration. Turning to GTC loops, (GTC)_14_ displays +1 and −1 slips, but in this case, these are indicative of triloops with populations of 8% and 17%, respectively. However, 60% of the time, the hairpin is in a zero slip or +2 slip (6%) configuration with a tetraloop; for (GTC)_15_, the data show slips of +1 (26%) and −1 (50%) with tetraloops and 26% with a zero slip with a triloop. Thus, both CAG and CTG hairpins predominantly slip by 2 TR units, jumping from tetraloop to tetraloop. GAC and GTC hairpins, on the other hand, slip primarily by a single unit jumping between tetraloops and triloops, respectively.

Turning to the structural results obtained from the MD simulations [124], it has been noted that for CTG, the 5′-CTG-3′ triloop resembles the 5′-CAG-3′ loop and is characterized by a sheared G-C pair held together by a single hydrogen bond. The triloops are subject to large fluctuations leading to an unraveling of the loop. By contrast, the 5′-TGCT-3′ tetraloop is much more stable and was observed to have either an anti–anti–anti–anti or an anti–syn–anti–anti conformation with differing stacking. The loop is stabilized by two hydrogen bonds between the T_anti_–T_anti_ mismatches and the flanking GpC step. The NMR studies agree with these results, showing that a structure based on four CTG repeats and clamped with a single GC pair gives rise to a stable hairpin with a TGCT loop [164].

For GTC, the simulations [124] indicate that the triloop 5′-GTC-3′ may exist in anti–anti–anti or syn–anti–anti conformations. However, unlike in the GAC triloop which has a sheared G_anti_–C_syn_ pair with two hydrogen bonds, there is no hydrogen bond between the G–C pair. The triloop is closed by an TC/GT step and therefore considerably weaker. The 5′-TCGT-3′ tetraloop is stabilized in two configurations: anti–anti–anti–anti and anti–anti–syn–anti. This loop is stabilized by two hydrogen bonds between the T_anti_–T_anti_ pair, which differs from that of the GAC tetraloop characterized by a single hydrogen bond between the A–A mismatched loop pairs. This feature, combined with the stacking arrangements, stabilizes the GTC tetraloop over the GAC counterpart.

smFRET investigations of CAG hairpins with CAA interrupts showed that there was a dramatic stabilization of slipped states when the G is mutated to A. For CTG, the corresponding experiments involve changing C to T, and so the effects of a TTG interrupt were examined [124]. This interrupt was placed near the loop, so that (CTG)_6_(TTG)(CTG)_8_, (CTG)_7_(TTG)(CTG)_7_ and (CTG)_8_(TTG)(CTG)_6_ hairpins were studied. For the latter two, switching is very much reduced over the original (CTG)_15_ hairpin. The (CTG)_7_(TTG)(CTG)_7_ hairpin is dominated by a single configuration at 0.54 FRET with a +1 slipped state, while (CTG)_8_(TTG)(CTG)_6_ has a state at 0.75 FRET consistent with both 0 and −1 slip states. These states all correspond to a tetraloop with the interrupting T being placed in the loop. The behavior of (CTG)_6_(TTG)(CTG)_8_ is somewhat more complex, but it too is consistent with a tetraloop configuration with the additional T placed in the loop [124].

### 2.3. Hairpins Formed from C and G Exclusive Trinucleotides

Here, we discuss hairpins formed from triplet repeat sequences containing only C and G. The most common examples are CGG and CCG TRs, which are overexpressed in the human genome. CGG TRs may be found in the 5′-UTR of the fragile X mental retardation gene (FMR1) [165]; similarly, TRs of CCG are also to be found in the 5′-UTR of several genes. The normal repeat range for CGG TRs is from about 5 to 54; a higher number of repeats leads to increased disease expression, causing fragile X syndrome (FXS) [166,167]. Specifically, a repeat length in the 55 to 200 range is associated with fragile X tremor ataxia syndrome (FXTAS) in males [168] and premature ovarian failure in females [169]. Longer repeats of 200 or more give rise to inherited fragile X mental retardation syndrome [170]. Likewise, CCG TRs are related to three TREDs, the longest expansion of which in the FRM2 gene gives rise to chromosome X-linked mental retardation (FRAXE) [171]. These repeats have also been implicated in HD and type 1 myotonic dystrophy [172].

#### 2.3.1. CGG, GGC, CCG and GCC Homoduplexes

The structural and dynamical characteristics of the C-rich and G-rich TR homoduplexes for DNA and RNA have been investigated, as these are characteristic of hairpin stems [118]. As with other TR-based duplexes, the properties of CGG (GGC) and CCG (GCC) TR hairpins are dominated by the behavior of G–G and C–C mismatches. Here, it is important to consider the Watson–Crick pairs surrounding the mismatches. Homoduplexes made from CGG and CCG are characterized by GpC steps between the mismatches, while CpG steps surround the mismatches in the GGC and GCC alignments. Free energy studies [118] show that the global minimum associated with the C–C mismatches in both DNA and RNA CCG or GCC correspond to the anti–anti configuration. Next, in terms of increasing free energy are anti–syn conformations followed by syn–syn. In this regard, the dihedral structure of the C–C mismatches is similar to the A–A and T–T (U–U for RNA mismatches in CAG/GAC and CTG/GTC TRs. However, the global free energy minimum associated with G–G mismatches within GGC and CGG TRs corresponds to the anti–syn conformation. In terms of the free energy, the next higher minimum corresponds to anti–anti, which is followed by syn–syn. We note that for RNA-CCG, it has been difficult to unambiguously resolve the actual ground state structure, as the anti–syn and anti–anti structures appear to degenerate within the limits of the calculations [118]. This difference appears to be due to the presence of a significant triple G-base stacking, which is not present in the other structures. Experimentally, there are crystallographic data for RNA CGG and CCG TRs—which have GpC steps between the mismatches—and these agree with the free energy results [155,173,174].

Direct MD simulations of the different homoduplexes in different conformations allow for the exploration of the transitions to the global minimum conformations [118]. On average, duplexes with C–C mismatches tend to transition to the anti–anti state faster than those with G–G mismatches. An examination of the MD runs shows that the additional stacking of the G-bases is what tends to slow down the transition. Another important feature is that for DNA GCC homoduplexes, the mismatched Cs may be extruded from the helical core to form a so-called “e-motif” discussed in the next section. Finally, a qualitative comparison of the relative stability of the mismatched homoduplexes via a fast laser melting technique [175] indicated that DNA ordering stability is as follows: G_rich_(CpG) > C_rich_(GpC) > G_rich_(GpC), which is in agreement with the currently available experimental data [118].

#### 2.3.2. E-Motif Formed by C- and G-Rich DNA Homoduplexes

**Figure 6:**
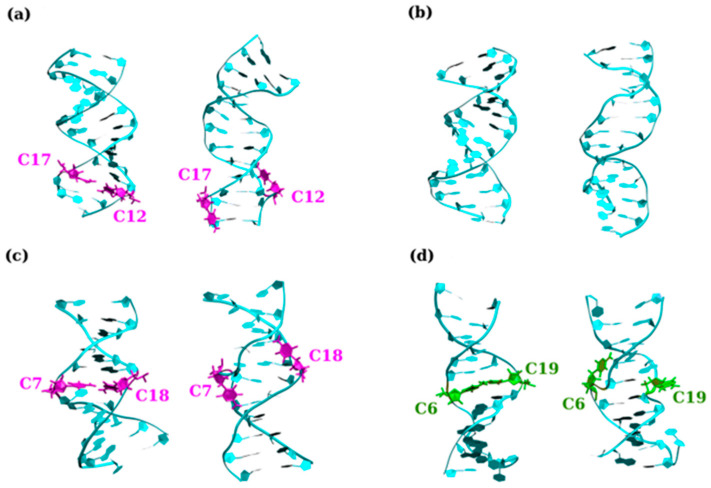
Sample configurations illustrating the e-motif as obtained with MD simulations. Initial (**left**) and final (**right**) structures are shown for (**a**) GCC4; (**b**) CCG4; (**c**) DC-1; and (**d**) DC-2. The C–C bases that ultimately form the e-motif are shown in purple. Bases shown in green are bases that are flipped out of the inner DNA helix but ultimately do not form an e-motif. See Ref. [122] for details.

In the last section, the structure and dynamical properties of homoduplexes with C–C and G–G mismatches are briefly summarized. It turns out, however, that the DNA homoduplexes can form structures that are considerably more complex by extruding the mismatches from their helical core in a well-defined manner to form the so-called “e-motif” [176], as shown in Figure 6. E-motifs were first discovered experimentally in an antiparallel DNA 5′-(CCG)_2_-3′ duplex via NMR (PDB 1NOQ). In this duplex, slippage conspired to form a central C–C mismatch surrounded by CpG Watson–Crick pairs [176]. These mismatched Cs flip out in the minor groove such that their base moieties point in the 5′ direction on each of the strands. See Figure 6 for sample e-motif configurations as well as contrasting nucleotides extruded from the helical core not in such a configuration. Since then, e-motif like structures have been observed in other C-rich DNA strands as well as other more complex structures such as an i-motif [177,178,179].

Theoretically, e-motif structures were observed to form during MD simulations of DNA d(CCCCGG) hexanucleotide repeats [114]. Motivated by these observations, other C-rich duplexes were subsequently examined in order to determine which sequences give rise to e-motifs either in an isolated or an extended form [122]. Specifically, the nonequivalent reading frames (GCC)_n_ and (CCG)_n_ for TRs and (CCCGGC)_n_, (CGGCCC)_n_ and (CCCCGG)_n_ for hexanucleotide repeats were examined. The results show for the basic e-motif, the C-bases of the ith residue in a mismatch, flip out in a symmetrical manner toward the i-2 residue (in the 5′ direction of each strand). The e-motif is stabilized by hydrogen bonds between the flipped out base and the i-2 base along the strand. This may be either a C-base (GCC) or a G-base for (CCCGGC). E-motif creation is made favorable through the creation of pseudo-GpC steps between non-adjacent base pairs when the C–C mismatches are extruded. As a result, the e-motif is stable in homoduplexes with GCC and CCCGGC but not in other reading frames [114]. It is interesting that an extended e-motif with all the mismatched Cs may also be formed. Such an arrangement is stabilized by the favorable stacking provided by the pseudo-GpC steps and the hydrogen bonds between the extruded Cs and the other nucleotides as well as a stacking between the Cs themselves. Ultimately, this results in a very stable but somewhat peculiar secondary structure.

While the e-motif structure formation is a feature of DNA GCC duplexes, it is not a characteristic of RNA-based duplexes. In our simulations, the C-bases in RNA duplexes were observed to flip out in a transient manner, but an e-motif was never formed. This is due to the presence of the additional hydroxyl group on RNA which tends to form hydrogen bonds with nearby sugars and the backbone. Additionally, the canonical A-form of RNA prevents good stacking for either GpC or CpG steps [118,122].

The e-motif is primarily associated with the extrusion of C-bases in B-DNA; it has recently been shown that e-like motifs are also associated with the extrusion of G-bases in left-handed Z-DNA, leading to the formation of a novel eGZ-motif [115]. Experimental CD spectroscopy experiments demonstrate that under high salt conditions, CGG may adopt a left-handed conformation [180,181]. MD simulations were used to examine all possible Z-DNA helices that could form after such a B- to Z-DNA transition [115]. It turns out that for such structures, the GG mismatch conformations may be arranged either intra- or extra-helical conformations, leading to the formation of BZ or ZZ junctions, or in an alternating extruded conformation. The most stable conformation is associated with alternately extruded Gs followed by a helix with symmetrically extruded Gs forming a ZZ junction. The extruded Gs in the left-handed helices are primarily in a syn conformation with favorable hydrogen bonds and stacking interactions, forming novel eGZ-motif structures [115].

#### 2.3.3. Structure and Dynamics of CCG and GGC Hairpins

CGG hairpins, both with and without AGG interrupts, have recently been studied with smFRET [182]. As with the hairpin structures already discussed above, the CGG hairpins exhibit even/odd parity behavior. It was found that CGG repeats predominantly form a so-called “blunt-end” structure consistent with a tetraloop structure. Thus, (CGG)_n_ with n-even is believed to form a 5′-GGCG-3′ tetraloop with no overhangs and a matched stem, while n-odd forms tetraloop 5′-GCGC-3′ with a single G overhang in the stem. There is also evidence for the formation of a small quadruplex population [182].

It is important to note that AGG interrupts in CGG TRs play an important role in suppressing FXS gene expression. AGG interrupts are found in most healthy individuals, occurring roughly every 9-10 CGG repeats [183], while those with a family history of FXS tend not to have such interrupts [184]. The recent mapping of CGG alleles shows that there is a strong effect of AGG interruptions on CGG TR expansion [185,186].

Given the importance of AGG in suppressing FXS disease expression, its effect was investigated by placing such interrupts in various location of a CGG-based hairpin. Placing the interrupt at the loop—i.e., (CGG)_9_AGG(CGG)_9_—leads to a dynamic reconfiguration of the loop from a 5′-GAGG-3′ loop with a single G overhang in the stem to a 5′-GGAG-3′ loop with three nucleotide GGC (i.e., one TR unit, from end) overhang. Since AGG interruptions in healthy individuals occur rather frequently, there are additional strands such as (CGG)_4_AGG(CGG)_n_AGG(CGG)_4_ with n standing for the number of CGG repeats separating the interrupts. For these kinds of strands, a somewhat modified parity-dependent behavior is observed. For odd-n, data are consistent with a tetraloop hairpin structure 5′-GGCG-3′ with a three-nucleotide overhang at one terminus. However, there now is an GGAG/GGAG bulge formed by the two AGG interrupts in the CGG hairpin stem. For n-even, a 5′-CGG-3′ triloop appears to be formed with a similar bulge at the two interrupts as well as a GGC overhang in the stem end. The exception is when *n* = 8 for which the flanking (CGG)_4_ participates to form a “dumbbell”-type structure with two GAGG tetraloops at both ends [187]. Thus, the presence of AGG interrupts favors the formation of the more stable 5′-GGAG-3′ tetraloop and/or the formation of stable loops within the CGG-based homoduplexes. These in turn are believed to restrict hairpin reconfiguration dynamics and inhibit TR repeat expansion.

## 3. Discussion

The structure and dynamics of trinucleotide repeat DNA hairpins are determined by a subtle balance between stem and loop interaction free energies. Single base changes arising from interrupts in the sequence pattern can have major impacts on these behaviors. The fact that these interruptions also have a substantial biological influence on disease-related expansion phenomena strongly suggests that the dynamics of trinucleotide repeat hairpins play a key role in these disease mechanisms [188].

Here, we have described recent progress using atomistic molecular dynamics simulations and smFRET experiments to characterize these atypical DNA structures and dynamics. Parity-dependent effects and strongly preferred slipping in one or two trinucleotide repeat units in different systems have been characterized. These reflect the preference for certain configurations of the loop that restrict stem alignment freedom. Different behaviors among distinct sequence variants highlight the impact of stacking energies for adjacent bases [11,117,118,119,120,121,122,123,124].

**Figure 7:**
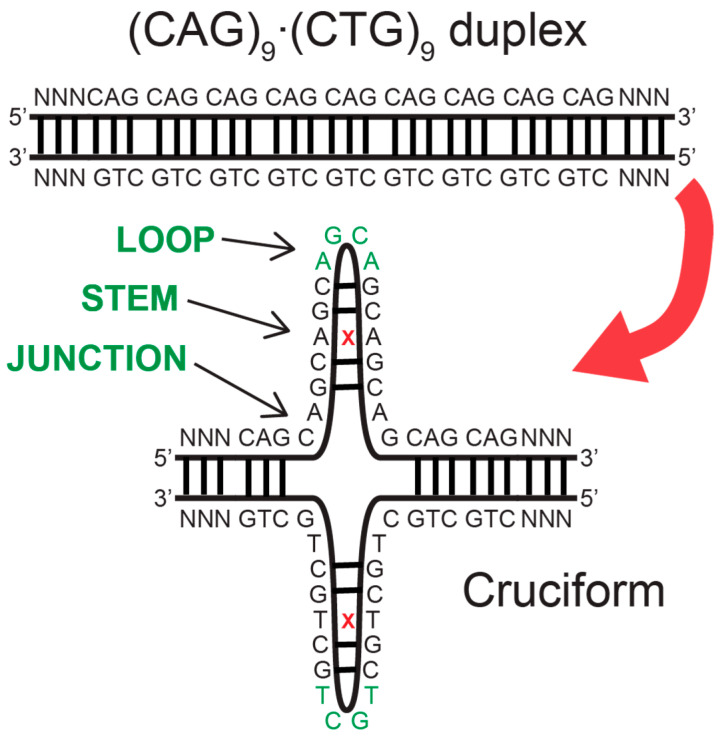
Schematic of a trinucleotide repeat containing duplex DNA (**upper**) opening into a cruciform arrangement (**lower**). The loop, stem, and junction region of the hairpin loop in the cruciform are indicated. The mismatched base pair in the stem is indicated by the red x. See Ref. [124] for more information.

These observations motivate models of how the dynamics of three-way junctions and cruciforms might contribute to trinucleotide expansions. See Figure 7 for a schematic. Genomic expansions likely involve the opening of the complementary duplex at some point during replication, transcription or repair-related processes. This duplex opening provides opportunities for hairpin-like structures to form from the single-stranded repeat regions folding on themselves. These hairpins would initially form opposite from each other in the opened duplex and have a high likelihood to collapse into each other, reforming the duplex. In some cases, the different slipping dynamics of the complementary sequences could allow them to migrate apart with different preferred directions or at different speeds [124,145]. Once they are isolated, they could persist sufficiently long to be engaged by the proteins that have been identified as involved in expansion phenomena. In these sorts of models, the dynamics of the hairpins are fundamental in determining the details of disease phenomena.

Structures formed from human disease-associated triplet repeats are dynamic with common themes being hairpins slipping in steps of multiples of the repeat unit. Strong evidence that the dynamics of these structures are significant for disease processes was recently provided by the discovery that a small molecule, naphthyridine–azaquinolone (NA), that binds to CAG hairpins, suppresses their slipping and also is curative for Huntington’s disease in mouse models [57]. Developing methods may provide windows into the formation of these structures inside living cells [189,190]. GWAS studies, transgenic mouse models, and other methods implicate several proteins, especially Fan1 [191,192,193,194,195,196] and mismatch repair (MMR) proteins in the mechanisms that lead to the disease-associated expansion of TNR in the human genome [76,197]. A critical frontier is discovering the interplay between the conformational dynamics of these atypical DNA structures and the proteins that are causative for expansion. Despite our lack of mechanistic understanding of how these proteins are involved, therapeutics for many of these diseases are already in development that pursue strategies to alter these protein–DNA interactions [198,199].

The largest family of proteins implicated in promoting TNR expansion is MMR proteins. DNA MMR’s primary function is to repair rare single base mismatches or short insertion/deletion loops that escape polymerase proofreading during replication [69,70]. The MutSα variant (MSH2-MSH6) targets the repair of mismatched bases, whereas the MutSβ variant (MSH2-MSH3) preferentially initiates the repair of loops [69,70]. The knock-out and GWAS data (above) implicate DNA binding activity of several proteins with most attention focusing on MutSβ and the DNA backbone nicking function of MutLγ, but the actual events resulting in expansion are unknown. Recent cryo-EM studies of MutSβ revealed multiple classes of conformations on short (CAG)_2_ loops and longer CAG hairpins [200,201]. These states inspired models of possible event sequences but have not yet incorporated any interaction with MutL variants.

Several experimental threads have suggested a spectrum of ideas about detailed mechanisms promoting expansions that include MutSβ-induced DNA bending [202] and the recruitment of distinct MutL variants [203]. A common theme in these models is a dynamic CAG hairpin that can change size and migrate [57,124,163,204]. Hairpin dynamics can generate asymmetric structures on the complementary strands of duplex DNA that can be differentially modified by MMR proteins. Precisely when and where the MMR proteins nick the DNA leads to distinct outcomes.

Currently, many therapeutic approaches are focused on affecting MSH3, which impacts MutSβ but not MutSα with the intent of maintaining most MMR activity [198,199,205]. Gene therapy and editing, antibodies and other biologics, as well as small molecules are all being developed and tested for impacts on TNR expansion disorders in cell and mouse models as well as in a few human trials [98,199,206,207]. Increased understanding of the dynamics of TNR hairpins and the impact on hairpin dynamics on the interactions they have with MMR proteins will enhance the targeting of these sorts of efforts to develop approaches to treat and prevent these diseases.

Determining how proteins promote somatic TNR expansion is challenging because (i) the precise TNR-containing DNA structure that recruits MMR proteins during expansion remains unconfirmed (with exciting new participants emerging occasionally [208]), (ii) the DNA structures that form and the DNA–protein interactions are dynamic and transient with a myriad of possible complexes possible, and (iii) these protein interactions are regulated by coordinated ATP dependencies that are difficult to synchronize in biochemical studies. Despite the difficulties, major advances will follow uncovering these DNA configurations, protein complexes, interactions, and ATPase regulatory mechanisms. Such efforts will be foundational to guide searches for TNR expansion disease therapeutics that seek to modify them to treat or prevent the disease.

## Figures and Tables

**Table 1:** A list of the locations on genes of the most common TRs along with an abbreviation of the most common diseases that are associated with them [11].

Location on Gene	Repeat	Disease
5′-UTR	CGG	FRAXA, FXTAS
	GCC	FRAXE
	CAG	SCA12
EXON	CAG	HD, HDL2, SBMA
		DRPLA, SCA1, SCA2, SCA3
		SCA6, SCA7, SCA17
	GAC	MSD
	GCG	SPD, HFG, ISSX, CCD
		HPES, OPMD, CCHS, BPES
INTRON	CAA	FRDA
	CCTG	DM2
	ATTCT	SCA10
	TGGAA	SCA31
	GGCCTG	SCA36
	GGGGCC	ALS
3′-UTR	CTG	DM1, HDL2, SCA8

## Data Availability

No new data were created for this review.
